# Venous Thromboembolism and Gut Dysbiosis: Mechanistic Links Between Endotoxemia, Microbial Metabolites, and Thromboinflammation

**DOI:** 10.3390/nu18081231

**Published:** 2026-04-14

**Authors:** Leon Smółka, Miłosz Strugała, Karolina Blady, Karolina Kursa, Bartosz Pomianowski, Agata Stanek

**Affiliations:** 1 Student Scientific Association, Department of Internal, Metabolic Diseases and Angiology, Faculty of Health Sciences, Medical University of Silesia, Ziolowa 45/47 St., 40-635 Katowice, Poland; s83152@365.sum.edu.pl (M.S.); s86570@365.sum.edu.pl (K.B.); s91506@365.sum.edu.pl (K.K.); s83086@365.sum.edu.pl (B.P.); 2 Department of Internal, Metabolic Diseases and Angiology, Faculty of Health Sciences, Medical University of Silesia, Ziolowa 45/47 St., 40-635 Katowice, Poland; 3 Upper Silesian Medical Center, Medical University of Silesia, Ziolowa 45/47 St., 40-635 Katowice, Poland

**Keywords:** venous thromboembolism, gut microbiome, endotoxemia, immunothrombosis, tissue factor, trimethylamine N-oxide (TMAO), neutrophil extracellular traps (NETs), thromboinflammation

## Abstract

**Background:** Venous thromboembolism (VTE), including deep vein thrombosis and pulmonary embolism, is increasingly recognized as a thromboinflammatory disorder involving coagulation, innate immunity, endothelial dysfunction, and vascular homeostasis. Emerging evidence suggests that gut microbiome-related inflammatory and metabolic signals may influence pathways potentially relevant to VTE through intestinal barrier dysfunction, microbial translocation, and microbiome-derived metabolites. This review critically examines the direct and indirect evidence relating gut dysbiosis to mechanisms potentially relevant to venous thrombogenesis. **Methods:** A structured literature search of PubMed, Scopus, and Web of Science was conducted from database inception to February 2026. Observational, translational, experimental, preclinical, and selected genetic studies were narratively synthesized across heterogeneous evidence types. **Results:** Available evidence suggests that intestinal barrier dysfunction and microbial translocation may increase systemic exposure to lipopolysaccharide and other microbial products, potentially contributing to inflammatory signaling and procoagulant responses. Proposed downstream effects include tissue factor (TF) activation, platelet reactivity, neutrophil extracellular traps (NETs) formation, complement signaling, endothelial perturbation, and impaired balance of anticoagulant and fibrinolytic pathways. Microbiome-derived metabolites, including trimethylamine N-oxide (TMAO), phenylacetylglutamine (PAGln), bile acids, and short-chain fatty acids (SCFAs), have been linked, mainly in experimental or non-VTE settings, to thrombosis-related biology. However, most evidence remains indirect, associative, or experimental, whereas direct human VTE-specific evidence is limited and heterogeneous. **Conclusions:** The gut microbiome–VTE axis is biologically plausible and supported mainly by mechanistic and indirect evidence, but current data are insufficient to support strong causal conclusions. Further longitudinal, well-phenotyped, mechanistically informed studies are needed to determine whether microbiome-related pathways have measurable clinical relevance in human VTE.

## 1. Introduction

### 1.1. Clinical Burden and Unmet Needs in Venous Thromboembolism

Venous thromboembolism (VTE), encompassing deep vein thrombosis (DVT) and pulmonary embolism (PE), represents a major global health burden with substantial morbidity, mortality, and healthcare costs [1,2]. Epidemiological analyses indicate that VTE affects approximately 1–2 per 1000 individuals annually in Western populations, with incidence increasing sharply with age [1,3,4]. Importantly, VTE is not merely an acute event, as it is frequently associated with recurrence and long-term sequelae. Cumulative recurrence rates approach 30% at 10 years after a first unprovoked event, despite appropriate anticoagulation [5,6,7].

PE remains an important cause of preventable in-hospital death [8,9,10], while long-term complications such as chronic thromboembolic pulmonary hypertension (CTEPH) and post-thrombotic syndrome (PTS) indicate that VTE may have sustained vascular and inflammatory consequences beyond the acute phase [11,12,13]. These observations support viewing VTE not only as a hemostatic disorder but also as a thromboinflammatory condition shaped by interactions among coagulation, inflammation, and endothelial dysfunction [14,15,16,17]. Elevated inflammatory biomarkers, including C-reactive protein and interleukin-6, have been associated with incident and recurrent VTE, suggesting that inflammatory pathways may be involved in the pathobiology of VTE [15,16,17,18].

Despite the availability of direct oral anticoagulants (DOACs), management strategies remain focused primarily on inhibition of thrombin or factor Xa [19,20]. While effective at reducing acute clot propagation, this approach does not directly target upstream endothelial and innate immune pathways and is limited by bleeding risk, underscoring the need to explore additional modulators of thromboinflammation.

Emerging evidence suggests that systemic inflammatory and metabolic perturbations may modulate venous thrombogenicity. Chronic inflammatory states—including obesity, inflammatory bowel disease (IBD), and malignancy—are associated with increased VTE risk, implicating immune dysregulation as one potential pathogenic axis [21,22,23,24]. Because vascular phenotypes vary across disease settings, interpretation of prothrombotic risk requires caution [25,26]. These observations raise the possibility that extravascular factors, including the gut microbiome and intestinal barrier integrity, may modulate systemic inflammatory and metabolic states relevant to thrombosis [27,28,29]. Simultaneously, direct evidence linking gut microbiome alterations specifically to human VTE remains limited, and much of the available literature derives from related inflammatory or cardiometabolic conditions, experimental models, or mechanistic studies rather than VTE-specific clinical investigations [30]. This distinction frames the microbiome–VTE relationship as an emerging and biologically plausible field rather than an established causal pathway.

### 1.2. Immunothrombosis as an Extension of Virchow’s Triad 

The classical Virchow’s triad—venous stasis, endothelial injury, and hypercoagulability—remains foundational to VTE pathogenesis. Contemporary concepts extend this framework by incorporating immunothrombosis, whereby innate immune responses contribute to intravascular coagulation and may become maladaptive in sterile inflammatory settings [31,32,33,34]. Mechanistically, tissue factor (TF) expression, platelet activation, neutrophil extracellular traps (NETs) formation, and complement signaling connect inflammation with coagulation and help frame VTE as a thromboinflammatory process [31,32,33,34,35,36,37,38,39,40,41]. Venous thrombosis differs from arterial thrombosis in its predominant fibrin- and red blood cell-rich composition, yet immune mechanisms remain important within this framework [31,35,42]. Within this framework, systemic drivers of innate immune activation may represent potential determinants of VTE risk [31,43]. Chronic exposure to pathogen-associated molecular patterns (PAMPs), including lipopolysaccharide (LPS), may promote procoagulant phenotypes in monocytes and endothelial cells, while low-grade endotoxemia has been associated with increased circulating TF-positive microparticles and enhanced thrombin generation, providing biological plausibility for a link between microbial signals and coagulation activation [41,43,44].

The gut microbiome has been proposed as one possible source of upstream inflammatory and metabolic signals relevant to this axis [10,45,46]. Dysbiosis-associated increases in intestinal permeability may facilitate systemic translocation of LPS and other microbial products, which could sustain innate immune activation and endothelial dysfunction [45,46,47]. However, these mechanisms are supported predominantly by indirect, translational, or experimental evidence rather than by direct VTE-specific human studies. This distinction is particularly relevant because evidence derived from arterial thrombosis, cardiometabolic disease, or broader inflammatory settings cannot be assumed to translate directly to venous thrombosis, given important pathophysiological differences between these conditions [25,26,31,35,42].

In this context, gut-derived inflammatory and metabolic signals may be considered as candidate upstream modulators of pathways potentially relevant to VTE, although direct clinical evidence remains limited. Against this background, the central question is not whether the gut microbiome has already been proven to drive VTE, but rather which microbiome-related pathways are currently supported by direct evidence, which remain indirect or hypothesis-generating, and where the major translational gaps persist. Therefore, the aim of this review is to critically examine the available literature linking gut dysbiosis, endotoxemia, and microbiome-derived metabolites with thromboinflammatory mechanisms relevant to VTE, while explicitly distinguishing biological plausibility from direct clinical evidence.

## 2. Materials and Methods

**Figure 1:**
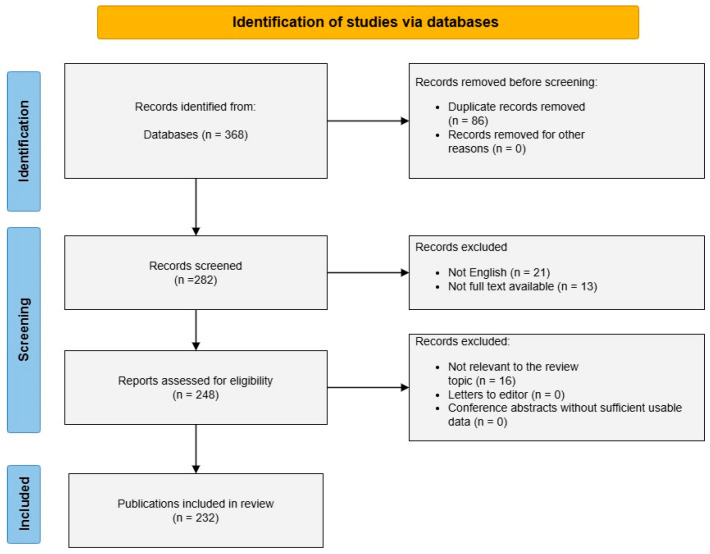
A flow diagram of study selection.

A structured literature search was conducted in PubMed, Scopus, and Web of Science from database inception to February 2026 to identify publications addressing associations between the gut microbiome and VTE, including DVT and PE, as well as related mechanisms involving intestinal barrier dysfunction, endotoxemia, microbiota-derived metabolites, immunothrombosis, and anticoagulant-related microbiome interactions. This broad search window was intentionally applied because direct human studies specifically examining the microbiome–VTE axis remain limited, and the available evidence base includes a substantial proportion of older mechanistic, translational, and foundational studies that continue to inform current concepts in this field. Accordingly, the presence of older citations reflects the fragmented and still limited nature of the recent VTE-specific evidence base, which constrains the strength and clinical interpretability of current conclusions. The search strategy combined terms related to the gut microbiome (e.g., gut microbiota/microbiome, dysbiosis, metagenomics, and microbial metabolites) with terms related to VTE and thromboinflammatory pathways (e.g., DVT, PE, thrombosis, coagulation, TF, thrombin, platelet activation, NETs, LPS, endotoxemia, gut barrier, intestinal permeability, TMAO, SCFAs, bile acids, anticoagulants, warfarin, and DOACs). Search terms were combined using Boolean operators (AND/OR) and adapted to each database’s syntax. The search was designed to capture both direct VTE-specific studies and indirect mechanistic literature relevant to thromboinflammatory pathways. The core PubMed-style search logic was based on the following concept blocks: (“gut microbiome” OR “gut microbiota” OR dysbiosis OR metagenomics OR “microbial metabolites” OR endotoxemia OR lipopolysaccharide OR “intestinal permeability” OR “gut barrier” OR trimethylamine N-oxide OR TMAO OR “short-chain fatty acids” OR SCFAs OR “bile acids”) AND (“venous thromboembolism” OR VTE OR “deep vein thrombosis” OR DVT OR “pulmonary embolism” OR PE OR thrombosis) AND (“tissue factor” OR thrombin OR “platelet activation” OR “neutrophil extracellular traps” OR NETs OR complement OR immunothrombosis OR coagulation). Comparable syntax-adapted strategies were then applied in Scopus and Web of Science using the same three concept domains: microbiome-related exposure terms, VTE-related outcome terms, and mechanistic thromboinflammatory terms. Additional combinations of synonyms and mechanism-specific terms were used iteratively across databases to improve the retrieval of both direct VTE-related studies and indirect literature relevant to endothelial activation, gut barrier dysfunction, microbial translocation, and anticoagulant-related interactions. Records were deduplicated before screening, and titles, abstracts, and full texts were assessed for eligibility. Screening was performed according to predefined eligibility criteria focused on relevance to the microbiome–VTE axis or to mechanistic pathways considered potentially relevant to VTE. Excluded were non-English publications, reports without accessible full text, conference abstracts without sufficient usable data, letters to the editor without relevant analytical content, and articles not relevant to the scope of the review. The full PubMed search strategy, including the exact search terms and limits used, is provided in Supplementary Method S1. The overall literature screening and study selection process across databases is summarized in Figure 1.

Eligible publications included original human, observational, translational, preclinical, and relevant genetic studies, as well as selected review articles used only to support contextual interpretation of the microbiome–thrombosis axis. Because direct VTE-specific human evidence remains sparse, studies of different designs were included to capture both clinically observed associations and biologically plausible mechanisms. However, these evidence categories were not considered equivalent and were interpreted according to study design, directness to VTE, and translational relevance. For interpretive clarity, direct human VTE-specific studies were considered the most clinically informative, followed by human studies in related thromboinflammatory conditions, genetic and multi-omics analyses, animal and in vitro mechanistic studies, and contextual review literature. Extracted data included the study design, population or experimental model, microbiome assessment method, key microbial taxa or functional pathways, metabolite-related findings, markers of gut permeability or endotoxemia, inflammatory and coagulation-related mediators, thrombotic outcomes, and major study limitations. The evidence was synthesized narratively and organized into thematic domains covering dysbiosis and pathobionts, intestinal barrier dysfunction and endotoxemia; microbiota-derived metabolites; chronic diseases associated with elevated VTE risk; and a dedicated section integrating direct human, genetic, and multi-omics evidence to contextualize mechanistic findings. No formal risk-of-bias or quality assessment tool was applied, because this was a narrative review integrating heterogeneous evidence types rather than a systematic review or meta-analysis. Instead, emphasis was placed on the critical interpretation of the literature, with attention to the strength, directness, and clinical relevance of the available evidence. Accordingly, findings were interpreted with explicit attention to evidence type, methodological heterogeneity, and the distinction between mechanistic plausibility and direct clinical relevance to VTE. This review should therefore be interpreted as a structured narrative synthesis of a heterogeneous, still-evolving evidence base, rather than as a quantitative assessment of a mature, methodologically uniform literature.

## 3. Pathogenetic Gut Bacteria and Dysbiotic Signatures in Chronic Diseases

### 3.1. Gram-Negative Pathobionts and LPS Structural Heterogeneity

This section summarizes predominantly mechanistic and contextual evidence relevant to dysbiosis-associated inflammatory signaling, while recognizing that direct VTE-specific human data remain limited. The intestinal microbiota includes abundant Gram-negative organisms whose outer membrane of LPS is a major trigger of innate immune signaling via Toll-like receptor 4 (TLR4) complexes [48,49]. Structural variation in the lipid A moiety influences the intensity of TLR4-mediated signaling, with some LPS species eliciting stronger proinflammatory responses than others [48,50,51].

Innate immune activation and coagulation are mechanistically intertwined, and LPS can induce TF expression on monocytes and endothelial cells [32,33]. This may favor thrombin generation and fibrin formation, while inflammatory signaling may also impair endogenous anticoagulant pathways such as the protein C system [32,52]. Experimental human endotoxemia models demonstrate that low-dose LPS exposure increases thrombin–antithrombin complexes and D-dimer concentrations, supporting the concept that endotoxin exposure can activate coagulation in vivo; however, these models do not provide direct evidence for a microbiome-specific mechanism in human VTE [43,44].

LPS-related inflammatory activation has also been linked to NET formation and TLR4-dependent venous thrombogenesis in experimental systems, including murine DVT models in which NET inhibition reduced thrombus burden [35,36,37,53,54,55]. These findings support biological plausibility but remain predominantly preclinical and should be interpreted as mechanistic rather than direct microbiome-specific evidence in human VTE. Taken together, these findings support mechanistic plausibility for LPS-related coagulation priming, but they do not establish a direct microbiome-specific causal pathway in human VTE [35,36,37,43,44,53,54,55].

### 3.2. Barrier Dysfunction and Microbial Translocation 

Intestinal barrier integrity depends on tight junction architecture that regulates paracellular permeability [45,56,57,58]. Proinflammatory cytokines and dietary perturbations can disrupt this barrier, increasing permeability and facilitating systemic translocation of LPS and other microbial products [45,57].

High-fat feeding has been shown in experimental models to elevate circulating LPS concentrations, a state often termed metabolic endotoxemia, which may precede obesity and insulin resistance in these settings [46,59]. Circulating biomarkers such as lipopolysaccharide-binding protein (LBP) are commonly used as indirect proxies for endotoxin exposure and have been associated with incident metabolic disease in population studies [47,60]. Barrier impairment is frequently observed in obesity and type 2 diabetes, conditions that are themselves independently associated with increased VTE risk [22,61]. Prospective cohort data further show that components of obesity and metabolic syndrome are associated with incident VTE, supporting a broader link between systemic inflammatory-metabolic perturbations and venous thrombotic risk rather than a direct microbiome-specific pathway [62,63,64,65,66]. These associations should be interpreted primarily as contextual metabolic-inflammatory support rather than as direct evidence for a microbiome-specific pathway in VTE.

Other metabolic conditions linked to dysbiosis, including metabolic dysfunction-associated steatotic liver disease (MASLD), may further broaden the spectrum of chronic inflammatory states in which endotoxemia, endothelial dysfunction, and hypercoagulability coexist [67,68]. Because MASLD is increasingly recognized as a dysbiosis-associated, proinflammatory condition with vascular and hemostatic perturbations, this provides additional contextual support for a thromboinflammatory milieu rather than for a direct microbiome-specific mechanism of VTE; dedicated reviews should be consulted for a more detailed discussion of the microbiome–MASLD axis [67,68].

Taken together, these observations support the relevance of barrier dysfunction and low-grade microbial translocation as components of a broader thromboinflammatory context, but direct evidence linking these processes to a microbiome-specific causal pathway in human VTE remains limited [22,46,47,59,60,61,62,63,64,65,66,67,68].

### 3.3. Chronic Diseases with Elevated VTE Risk as Dysbiosis-Associated States 

IBD is associated with a significantly increased risk of VTE, particularly during periods of active inflammation [23,69]. IBD is characterized by dysbiosis and epithelial barrier disruption, features that may contribute to systemic immune activation and procoagulant signaling; however, their specific contribution to VTE should be interpreted as contextual and inferential rather than as directly demonstrated microbiome-specific evidence [70,71,72].

Malignancy represents another high-risk state for VTE, with validated clinical prediction models demonstrating substantial thrombosis incidence during chemotherapy [73,74]. Cancer-associated systemic inflammation and TF expression can amplify coagulation pathways and are thought to contribute to venous thrombogenesis, but in the present context, these observations should be viewed as supportive of a broader inflammatory-thrombotic framework rather than as direct evidence for a microbiome-specific mechanism [24,75].

Collectively, dysbiosis, barrier dysfunction, endotoxemia, and innate immune activation may represent overlapping biological features across chronic inflammatory and metabolic disorders associated with elevated VTE risk. These observations provide contextual support for a broader thromboinflammatory framework in which gut-derived immune signaling may be relevant, but they do not show that microbiome alterations independently drive VTE [27,43,46]. Table 1 summarizes the main dysbiosis-related triggers and barrier mechanisms discussed in Section 3.1, Section 3.2 and Section 3.3, and indicates the varying types and levels of evidence supporting these proposed links.

Taken together, these disease associations support biological plausibility and contextual relevance, but they do not isolate the microbiome as an independent causal determinant of VTE in humans [23,24,27,43,46,69,70,71,73,74,75].

Integrative interpretation of the studies summarized in Table 1 suggests a conceptual thromboinflammatory framework linking dysbiosis, barrier dysfunction, endotoxemia, and innate immune activation to pathways potentially relevant to elevated VTE risk; however, the directness and strength of the evidence vary substantially across entries [27,43,45,46].

## 4. Endotoxemia and Tissue Factor Activation

### 4.1. LPS–TLR4–NF-κB–Tissue Factor Axis 

LPS, a major structural component of the outer membrane of Gram-negative bacteria, may enter systemic circulation following translocation from the gastrointestinal tract. This process is facilitated by increased intestinal permeability, which may develop in the setting of intestinal inflammation, gut microbiota dysbiosis, and exposure to drugs that alter microbial composition, including antibiotics [76,77]. As a result, gut-derived endotoxemia has been proposed as a biologically plausible link between dysbiosis, chronic low-grade inflammation, and vascular disease, although direct VTE-specific human evidence remains limited.

In mechanistic and translational settings, LPS may contribute to a prothrombotic milieu. Experimental and translational evidence indicates that endotoxin can promote coagulation activation, platelet reactivity, and thromboinflammatory signaling, processes that may be relevant to vascular complications [78,79]. Some of these effects have been described even in the absence of overt structural endothelial injury, suggesting that endotoxin exposure may promote a procoagulant state without gross endothelial disruption. At the molecular level, LPS signals through LBP, CD14, and the TLR4–myeloid differentiation protein 2 (TLR4–MD-2) complex, activating inflammatory transcriptional programs that can induce TF and other procoagulant mediators [80,81]. Downstream signaling through myeloid differentiation primary response 88 (MyD88)-, TIR-domain-containing adapter-inducing interferon-β (TRIF)-, nuclear factor kappa B (NF-κB)-, and interferon regulatory factor 3 (IRF3)-related pathways has been implicated in these responses [80,82]. Inflammatory stimulation in monocytes and TF-bearing circulating microparticles can increase TF activity and support thrombin generation [33,83]. TF overexpression by malignant cells illustrates the procoagulant importance of this pathway in a related disease context, but does not constitute microbiome-specific evidence for VTE [84,85].

Once activated, the TF pathway promotes thrombin generation, fibrin formation, and platelet activation [86,87,88]. Importantly, several of the coagulation-related effects of endotoxin are best established in acute inflammatory states and experimental endotoxemia models, whereas extrapolation to chronic low-grade endotoxemia in human VTE remains more inferential [33,77,78,79,83]. Taken together, these mechanisms provide a biologically plausible rationale for how endotoxin-triggered TF signaling could contribute to thromboinflammatory and procoagulant states. However, much of this evidence derives from mechanistic, experimental, or related-disease settings rather than direct studies of chronic low-grade endotoxemia in human VTE. Accordingly, this pathway should be interpreted as biologically plausible and experimentally supported, but not yet established as a microbiome-specific causal mechanism in VTE [33,77,78,79,83,86,87,88].

### 4.2. Impairment of Anticoagulant and Fibrinolytic Systems

Chronic inflammation associated with gut dysbiosis may promote not only TF-dependent coagulation, but also dysregulation of endogenous anticoagulant and fibrinolytic pathways. In this context, the mechanisms discussed below should likewise be interpreted primarily as indirect, mechanistically supported, rather than as direct evidence in human VTE [33,89]. Endotoxemia is associated with increased concentrations of proinflammatory cytokines, especially tumor necrosis factor alpha (TNF-α), interleukin 6 (IL-6), and interleukin 8 (IL-8), which contribute to endothelial activation, thrombin generation, and suppression of physiological hemostatic control mechanisms [33,89].

One important target of inflammatory dysregulation is antithrombin, a key natural anticoagulant. Under physiological conditions, antithrombin neutralizes thrombin and factor Xa; however, during systemic inflammation, its activity may decline due to reduced synthesis, increased consumption, proteolytic degradation, and impaired endothelial support [90,91]. Inflammation may also be associated with reduced hepatic synthesis of antithrombin as part of broader acute-phase shifts in liver protein production, although this pattern is most strongly supported in acute inflammatory settings and is more inferential when extended to chronic low-grade endotoxemia [90,92]. Activated neutrophils may further promote thrombogenicity through proteolytic degradation of anticoagulant factors and NET-related effects on clot architecture [91,93]. Increased vascular permeability may further lower effective intravascular antithrombin concentrations by facilitating transvascular leakage [94]. Together, these changes may weaken antithrombin-mediated inhibition of coagulation and favor hypercoagulability.

Inflammation may also impair the protein C anticoagulant pathway by reducing thrombomodulin and endothelial protein C receptor (EPCR) expression, thereby limiting activated protein C (APC) generation and weakening endogenous anticoagulant control [95,96,97,98]. Because APC also exerts anti-inflammatory and barrier-protective effects, impairment of this pathway may amplify both inflammation and coagulation [99,100,101,102].

Chronic inflammation is also associated with suppression of fibrinolysis. A central mediator of this process is plasminogen activator inhibitor 1 (PAI-1), the principal inhibitor of tissue-type plasminogen activator (tPA) and urokinase-type plasminogen activator (uPA). By reducing plasmin generation, elevated PAI-1 may impair fibrin degradation [103,104]. PAI-1 expression is stimulated by inflammatory cytokines, LPS, oxidative stress, and endothelial activation, making it a prominent feature of thromboinflammatory states [105,106]. This phenomenon is particularly well established in sepsis and disseminated intravascular coagulation, where impaired fibrinolytic capacity favors persistence of fibrin deposits and microthrombi; its relevance to chronic low-grade endotoxemia is therefore more inferential than directly demonstrated [105,106,107,108,109]. Accordingly, increased PAI-1 may represent one mechanism by which inflammation linked to dysbiosis or endotoxemia could contribute to impaired fibrinolysis and delayed thrombus resolution [109].

Another potentially relevant but largely mechanistic contributor is degradation of the endothelial glycocalyx, a surface layer that supports vascular barrier function, limits platelet and leukocyte adhesion, and helps maintain anticoagulant and anti-inflammatory signaling [110,111]. Inflammatory glycocalyx degradation may increase endothelial permeability, facilitate cellular adhesion, disrupt antithrombin- and protein C-dependent anticoagulant mechanisms, and create a more permissive surface for the assembly of coagulation enzyme complexes [112,113]. Because the endothelial glycocalyx regulates vascular barrier integrity, leukocyte adhesion, and anticoagulant surface properties, its degradation may contribute to a more proinflammatory and procoagulant endothelial phenotype; however, in the context of microbiome-related low-grade endotoxemia and VTE, this link remains largely indirect, and readers are referred to dedicated reviews for a more detailed discussion [110,111,112,113].

**Figure 2:**
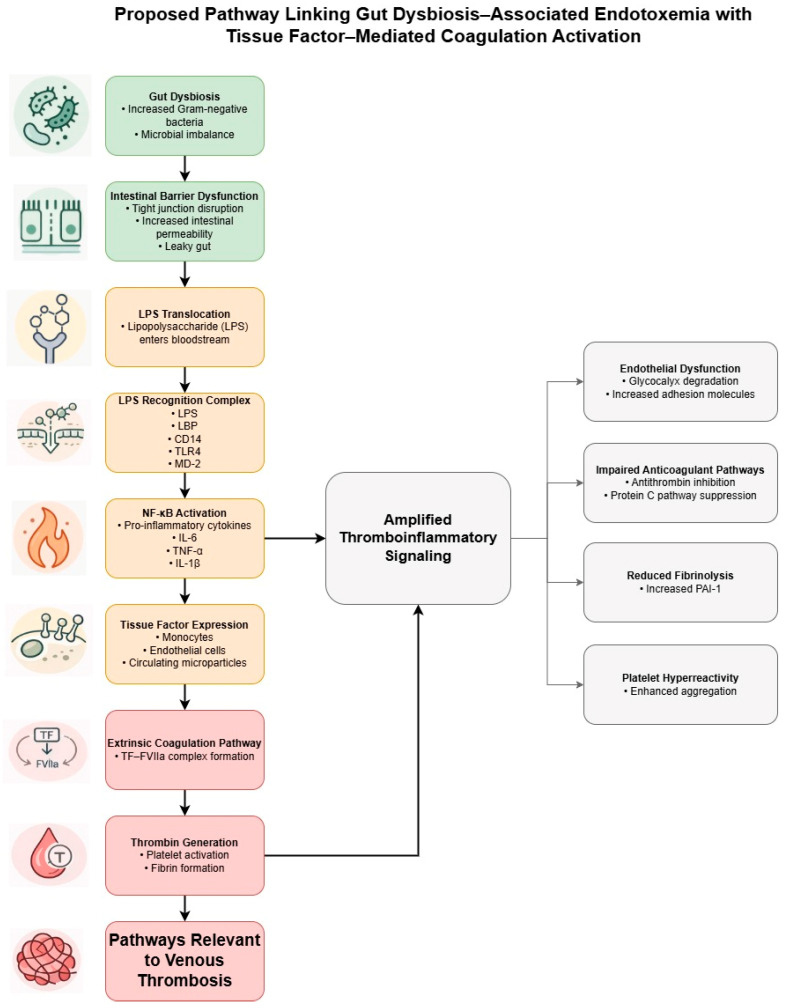
Proposed pathway linking gut dysbiosis-associated endotoxemia with TF-mediated coagulation activation and thromboinflammatory processes relevant to venous thrombosis. Gut dysbiosis and intestinal barrier dysfunction may increase intestinal permeability, facilitating LPS translocation into circulation. LPS is then recognized by the LBP-CD14-TLR4-MD-2 complex, which can activate NF-κB signaling and proinflammatory cytokine release. This response may induce TF expression in monocytes, endothelial cells, and circulating microparticles, thereby contributing to activation of the extrinsic coagulation pathway and thrombin generation, with downstream effects potentially relevant to venous thrombosis. In parallel, amplified thromboinflammatory signaling may contribute to endothelial dysfunction, impaired anticoagulant pathways, reduced fibrinolysis, and platelet hyperreactivity, thereby reinforcing a prothrombotic state. Several of these amplifying mechanisms are supported predominantly by mechanistic, experimental, or acute inflammatory models rather than direct evidence in chronic low-grade endotoxemia associated with human VTE. Green boxes indicate gut microbial and barrier-related processes, orange boxes represent inflammatory signaling, red boxes denote coagulation-related processes, and gray boxes show parallel amplifying mechanisms. Abbreviations: CD14—cluster of differentiation 14; FVIIa—activated factor VII; IL-1β—interleukin 1 beta; IL-6—interleukin 6; LBP—lipopolysaccharide-binding protein; LPS—lipopolysaccharide; MD-2—myeloid differentiation protein 2; NF-κB—nuclear factor kappa B; PAI-1—plasminogen activator inhibitor 1; TF—tissue factor; TF–FVIIa—tissue factor-activated factor VIIa complex; TLR4—Toll-like receptor 4; TNF-α—tumor necrosis factor alpha.

Taken together, these mechanisms suggest that endotoxemia-associated inflammation may promote hypercoagulability not only through activation of the LPS–TLR4–NF-κB–TF axis, but also through concomitant suppression of anticoagulant defenses and fibrinolysis. However, much of the supporting evidence derives from sepsis, disseminated intravascular coagulation, experimental endotoxemia, or related inflammatory settings rather than direct studies of chronic low-grade endotoxemia in human VTE. This integrated model therefore highlights biological plausibility rather than an established microbiome-specific mechanism of thromboembolic risk. A proposed pathway linking gut dysbiosis with TF-mediated coagulation activation and thromboinflammation is summarized in Figure 2.

## 5. Microbiome-Derived Metabolites Converging on a Prothrombotic Milieu

### 5.1. TMAO and Platelet Hyperreactivity 

TMAO is generated through a meta-organismal pathway in which gut microbiota metabolize dietary choline, phosphatidylcholine, and L-carnitine into trimethylamine (TMA), which is subsequently oxidized in the liver by flavin monooxygenases to TMAO [114,115]. Direct human evidence linking circulating TMAO to VTE remains limited; one of the few direct VTE-specific human studies reported no significant association with recurrent VTE, indicating that the clinical relevance in venous thrombosis remains uncertain [116]. In experimental systems, TMAO has been shown to enhance platelet responsiveness by amplifying agonist-induced intracellular calcium release, thereby lowering the threshold for platelet activation [117,118]. In murine thrombosis models, dietary choline supplementation increased plasma TMAO levels and accelerated thrombus formation, whereas suppression of gut microbial TMA production attenuated thrombosis, supporting a mechanistic role for TMAO-related pathways in experimental thrombosis rather than direct evidence in human VTE [117,118].

TMAO has also been reported to enhance TF expression and promote inflammatory signaling in vascular cells, suggesting a possible mechanistic link between metabolic dysbiosis and thromboinflammation in non-VTE settings [119]. In large and prospective clinical cohorts, elevated circulating TMAO concentrations have been associated with increased cardiovascular risk, supporting systemic biological relevance primarily in arterial and cardiometabolic settings rather than in VTE-specific populations [114,115,120]. Accordingly, platelet hyperreactivity and related thrombosis-associated effects should be interpreted as supporting broader thrombosis-related biology rather than as direct evidence of VTE-specific clinical relevance. Taken together, the strongest supportive data for TMAO derive from experimental models, platelet biology, and arterial cardiovascular cohorts rather than from VTE-specific populations. These findings support biological plausibility, but direct human evidence in VTE remains limited and inconsistent, including at least one clinically relevant null study, so extrapolation from atherothrombosis to venous thrombosis should be made cautiously [114,115,116,117,118,119,120,121].

### 5.2. Short-Chain Fatty Acids (SCFAs) 

SCFAs, primarily acetate, propionate, and butyrate, are generated through bacterial fermentation of dietary fiber and exert broad immunomodulatory effects [122]. SCFAs regulate immune responses through histone deacetylase (HDAC) inhibition and G-protein-coupled receptor signaling, promoting anti-inflammatory and barrier-supportive effects in experimental systems [123].

In experimental models, butyrate has been shown to enhance epithelial barrier integrity and suppress NF-κB activation, thereby limiting systemic inflammatory tone [124]. SCFAs have also been shown in experimental and cardiovascular settings to improve endothelial function and reduce leukocyte adhesion, mechanisms that may be relevant to thromboinflammatory modulation but have not been directly established in VTE-specific populations [125]. Experimental data indicate that SCFAs can modulate platelet function and thrombosis-related processes indirectly via anti-inflammatory signaling pathways in preclinical settings [122,126,127]. Taken together, these observations support a biologically plausible anti-inflammatory and endothelial-protective role for SCFAs, but their relevance to venous thrombosis remains indirect and hypothesis-generating rather than clinically established.

### 5.3. Bile Acids and FXR/TGR5 Signaling 

Gut microbiota convert primary bile acids into secondary bile acids, reshaping the bile acid pool and influencing host signaling through farnesoid X receptor (FXR) and Takeda G-protein-coupled receptor 5 (TGR5) pathways [128]. FXR signaling regulates lipid metabolism, inflammation, and endothelial function, and thus may indirectly influence pathways relevant to thromboinflammatory homeostasis in mechanistic and metabolic disease settings [129].

**Figure 3:**
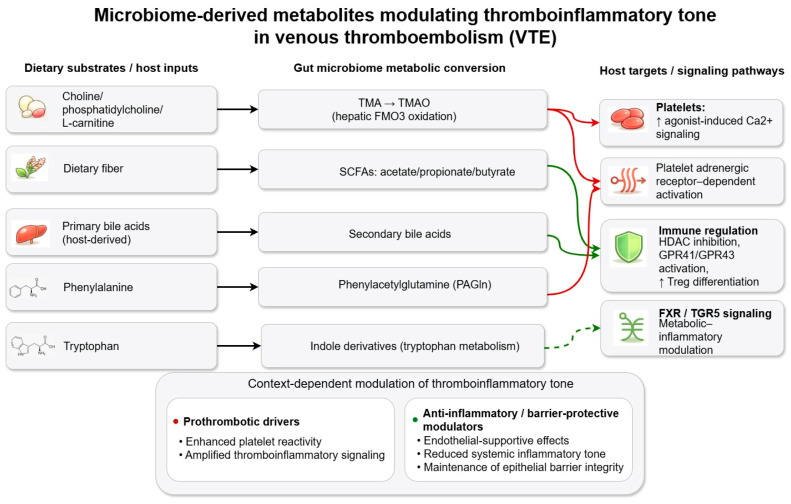
Proposed metabolite pathways by which microbiome-derived signals may influence thromboinflammatory tone relevant to VTE. The schematic summarizes proposed links between dietary substrates, microbiome-derived metabolites, and downstream host targets, highlighting platelet-related pathways with prothrombotic potential (red) and anti-inflammatory or barrier-protective pathways (green), whereas dashed arrows indicate more indirect or hypothesis-generating links. Abbreviations: Ca^2+^—calcium; FXR—farnesoid X receptor; FMO3—flavin-containing monooxygenase 3; GPR—G-protein-coupled receptor; HDAC—histone deacetylase; PAGln—phenylacetylglutamine; SCFAs—short-chain fatty acids; TGR5—Takeda G-protein-coupled receptor 5; TMA—trimethylamine; TMAO—trimethylamine N-oxide; Treg—regulatory T cell; VTE—venous thromboembolism.

Altered bile acid signaling in dysbiosis may indirectly influence systemic inflammatory tone and coagulation-related pathways through metabolic and endothelial mechanisms, although direct evidence of relevance to VTE remains limited [129,130,131]. Most available support for this pathway derives from mechanistic, metabolic, or hepatobiliary studies rather than from VTE-focused human investigations. Currently, this pathway is best viewed as contextual metabolic–inflammatory support rather than VTE-specific evidence. Figure 3 summarizes the major microbiome-derived metabolite axes discussed in Section 5.1, Section 5.2, Section 5.3 and Section 5.4 and highlights their predominantly experimental, indirect, or hypothesis-generating links to thromboinflammatory targets.

### 5.4. Emerging Metabolites (PAGln, Indoles) 

PAGln, a gut microbial metabolite derived from phenylalanine metabolism, has been shown in experimental and cardiovascular settings to enhance platelet responsiveness via adrenergic receptor-dependent pathways, but direct evidence for relevance to VTE remains limited [132,133]. In murine models, PAGln potentiated platelet aggregation and thrombosis, supporting the relevance of preclinical thrombosis rather than direct evidence in human VTE populations [132,133].

Indole derivatives produced from tryptophan metabolism modulate mucosal immunity and epithelial barrier integrity through aryl hydrocarbon receptor (AhR)-related signaling [134,135]. Through regulation of immune homeostasis and barrier stability, indole metabolites may indirectly influence pathways relevant to thromboinflammatory risk by modulating systemic endotoxemia and inflammatory activation; however, this link remains indirect and largely hypothesis-generating rather than directly established in VTE [134,135,136]. Table 2 summarizes the microbiome-derived metabolites discussed above (TMAO, SCFAs, bile acids, PAGln, and indole derivatives), including their precursor pathways, proposed host signaling targets, and the varying strength and directness of supporting evidence across study types. Importantly, the strength and direct relevance of this evidence to VTE vary substantially, with several pathways supported predominantly by mechanistic or non-VTE data [126,137].

## 6. NETs and Complement Amplification

NETs are recognized components of venous thrombi and of the broader immunothrombotic response in VTE; however, their placement within a gut microbiome–VTE axis remains much more indirect than their general relevance to venous thrombosis itself. These extracellular web-like structures are released by activated neutrophils in response to pathogens, inflammatory stimuli, and danger signals. Under physiological conditions, NET formation contributes to host defense, whereas in pathological settings, NET-associated components—including neutrophil elastase, myeloperoxidase, histones, and cathepsin G—may support procoagulant and proinflammatory responses relevant to thrombus formation [138,139].

NETs provide a mechanistically plausible link between inflammation and venous thrombosis. Inflammatory stimuli, including LPS and other pathogen-associated molecular patterns, may prime neutrophils to release NETs, thereby supporting a biologically plausible mechanism by which microbiome-related immune activation could intersect with thromboinflammatory pathways relevant to VTE [35,140]. However, direct evidence that chronic microbiome-related exposure drives NET-dependent thrombosis in human VTE remains limited. NETs can provide a scaffold for platelet adhesion and coagulation factor accumulation, while NET-associated histones and DNA may enhance endothelial injury, platelet activation, and contact pathway signaling in experimental settings [141,142].

Complement activation may further amplify thromboinflammation by promoting neutrophil, platelet, and monocyte activation; enhancing TF expression; and reinforcing NET formation; however, the strongest support for this axis comes from the broader literature on immunothrombosis and complement-mediated thromboinflammation rather than from direct studies showing that microbiome perturbations activate this pathway in human VTE [143,144,145,146,147,148].

Platelet–neutrophil crosstalk, including P-selectin-dependent interactions, may further reinforce NET-associated immunothrombotic responses in venous thrombosis [149,150,151]. NET-rich thrombi may also be relatively resistant to fibrinolysis because DNA and histones interact with fibrin, favoring a denser clot architecture. Consistent with this, circulating markers of NET formation, including cell-free DNA and histone-DNA complexes, have been associated with VTE, although their clinical utility as standalone diagnostic biomarkers remains limited, and these associations do not establish an upstream microbiome-specific mechanism [152,153].

NETs may also support TF expression and activity, thereby linking innate immune activation with extrinsic coagulation in experimental systems [154,155].

Taken together, NETs and complement are highly relevant to immunothrombotic amplification in VTE, but their incorporation into a gut microbiome–VTE model remains supported predominantly by mechanistic, experimental, and indirect inflammatory evidence rather than by direct microbiome-specific studies in human VTE. Thus, this pathway is best viewed as a biologically plausible amplifier of venous thromboinflammation, not as proof of a microbiome-specific causal mechanism [138,144].

## 7. Human, Genetic, and Multi-Omics Evidence

Human evidence linking the gut microbiome or microbiome-derived metabolites to VTE is still sparse [30,116,156,157,158,159,160,161,162]. Among the few VTE-specific studies available, Reiner et al. reported no significant association between TMAO and recurrent VTE, underscoring the still limited and non-uniform nature of the current clinical evidence [116]. Beyond VTE-specific cohorts, TMAO remains one of the most extensively studied microbiome-derived metabolites in thrombosis-related research; however, observational and experimental findings from non-VTE settings should be interpreted as contextual rather than direct evidence for venous thrombotic risk [114,115]. Because the mechanistic and cardiovascular literature on TMAO has been discussed above, the present section focuses primarily on how direct human VTE data compare with genetic and integrative lines of evidence.

Genetic approaches, particularly Mendelian randomization (MR), provide additional hypothesis-generating inference regarding possible links between gut microbiota composition, microbial metabolites, and thromboembolic phenotypes. By using genetic variants as instrumental variables, MR analyses can reduce susceptibility to some forms of confounding typical of observational studies; however, in the microbiome field, these analyses often rely on genome-wide association study (GWAS)-derived instruments with limited statistical power and taxonomic resolution, and their conclusions remain strongly dependent on instrument validity and modeling assumptions. In addition, the core MR assumptions—relevance, independence, and exclusion restriction—are difficult to fully verify in this setting, so these findings should not be interpreted as robust evidence of causality. One such analysis, performed by Cheng et al., investigated potential causal relationships among bacterial taxa, microbiome-derived metabolites, and VTE risk [163]. More broadly, microbiome-related cardiovascular and thromboinflammatory signaling has also been discussed in the wider literature, although such data should be interpreted as contextual rather than VTE-specific evidence [164]. In this analysis, genetically predicted bacterial taxa and microbial metabolites showed associations with estimated VTE risk in MR models. Lower genetically predicted abundance of several SCFA-related taxa, including members of *Firmicutes*, was associated with higher estimated thromboembolic risk, whereas other taxa showed inverse associations; these findings remain inferential rather than definitive biological evidence. The authors also discussed the possibility that microbiome-related inflammatory signaling may contribute to thrombotic phenotypes by affecting vascular and immune pathways [163]. In a separate mediation MR analysis, immune cell populations were proposed as potential intermediates linking gut microbiota-related traits with VTE risk, raising the possibility that microbiome-related effects on thrombosis may involve immune regulation; however, these findings remain model-based and require biological validation [30].

Another MR analysis using GWAS data for 207 bacterial taxa identified multiple taxa associated with estimated VTE risk, with several showing putative causal signals in Bayesian-weighted analyses [30,165]. An MR study conducted by Huang et al. further classified specific microbial taxa according to their inferred positive or negative associations with VTE risk [165,166]. In this analysis, genetically predicted abundance of several taxa within *Firmicutes* and *Actinobacteria* showed inverse or positive associations with estimated VTE risk, whereas other taxa, including *Lactobacillales* and *Lactococcus*, showed associations in the opposite direction. Given the exploratory nature of current microbiome GWAS instruments, these taxon-level findings should be interpreted cautiously.

Similarly, an MR analysis by Cen et al. investigated associations between gut microbiome composition and the risk of pulmonary embolism [165,167]. Using inverse-variance weighting, the authors identified several genera—including *Slackia*, *Oscillospira*, *Bacteroides*, and *Clostridium sensu stricto 1*—that showed inverse associations with estimated pulmonary embolism risk in the analyzed dataset [165,167].

Another two-sample MR analysis reported that higher genetically predicted abundance of taxa such as *Slackia*, *Butyricicoccus*, *Eubacterium coprostanoligenes* group, and *Bacteroides* was associated with lower estimated VTE risk, whereas increased abundance of *Coprococcus 1* was associated with higher risk [165,168,169]. Across these MR studies, the direction of association is not fully consistent across analyses, including within broadly defined groups such as *Firmicutes* [30,163,165,166,167,168,169]. This heterogeneity may reflect weak instruments, differences in taxonomic aggregation, population- and model-specific effects, and unresolved pleiotropy rather than stable, VTE-specific biological signals.

Currently, truly integrated VTE-specific host–microbiome multi-omics datasets remain very limited. Overall, the available human and genetic evidence supports continued investigation of a possible microbiome–VTE relationship, but it does not yet establish a consistent or causally defined signal. Importantly, any relationship between the microbiome and thrombotic risk is unlikely to depend on a single metabolite; broader microbiome-associated effects on immune and inflammatory signaling may also be relevant, although their relative contribution to VTE remains unresolved. Currently, such integrative interpretations remain largely exploratory rather than derived from validated VTE-specific host–microbiome datasets, and should therefore be viewed as a framework for future study rather than direct clinical evidence [30,170,171].

## 8. Microbiome–Anticoagulant Interactions

Oral anticoagulants remain the cornerstone of VTE treatment and secondary prevention, yet clinically meaningful interindividual variability persists despite standardized dosing algorithms and monitoring strategies [172]. In addition to established drivers (dietary vitamin K intake, drug–drug interactions, and host genetic determinants), the gut microbiome has been proposed as a potential modulator of anticoagulant response through effects on vitamin K homeostasis and intestinal xenobiotic handling [173]. Microbiome–drug relationships are bidirectional—medications can reshape microbial communities, and microbial functions can influence drug exposure—forming the conceptual basis of “pharmacomicrobiomics” [174]. However, the strength and clinical directness of evidence differ substantially across anticoagulant classes, with more practically relevant data available for warfarin than for direct oral anticoagulants (DOACs).

### 8.1. Warfarin: Microbiome-Driven Vitamin K Availability and International Normalized Ratio (INR) Variability 

Warfarin inhibits the vitamin K epoxide reductase complex, thereby limiting γ-carboxylation of vitamin K-dependent coagulation factors and rendering INR stability sensitive to fluctuations in vitamin K availability [171]. Beyond dietary phylloquinone (vitamin K1), menaquinones (vitamin K2 forms) are synthesized by intestinal bacteria and may contribute, to varying degrees, to host vitamin K pools [175]. Earlier work described bacterial menaquinone production and its potential relevance to coagulation homeostasis [176]. Human data further indicate that bacterially derived menaquinone can be absorbed in the distal intestine, providing a mechanistic pathway by which microbiome alterations might contribute to warfarin dose requirements and INR variability [177]. Even so, the magnitude of this contribution in routine clinical anticoagulation remains difficult to quantify because dietary intake, concomitant medications, illness, and host genetics also substantially affect INR stability.

### 8.2. Antibiotic-Associated Dysbiosis: Clinical Evidence of Warfarin Destabilization and Bleeding Risk 

Broad-spectrum antibiotic exposure has been associated with reduced menaquinone concentrations in human liver, supporting the concept that antimicrobial therapy can deplete microbiota-derived vitamin K2 reserves [178]. Such depletion provides biologic plausibility for increased warfarin sensitivity during antibiotic exposure, although clinical INR changes are multifactorial and may also reflect classic pharmacokinetic interactions and intercurrent illness [173]. Observational studies demonstrate that several commonly used antibiotics are associated with increased risk of overanticoagulation in warfarin-treated outpatients, with agent-specific variability [179]. Drug–drug interactions are also a frequent contributor to over-anticoagulation in VKA-treated (vitamin K antagonist-treated) cohorts, underscoring the clinical reality that INR instability often reflects multiple concurrent determinants [180]. Accordingly, antibiotic-associated warfarin destabilization is well recognized clinically, but the specific contribution of microbiome disruption is difficult to disentangle from conventional interaction mechanisms.

Agent-level evidence includes classic reports of potentiation with sulfonamide combinations, illustrating that clinically significant INR excursions can occur in otherwise stable patients [181]. Case reports also describe marked INR elevations after macrolides such as azithromycin, reinforcing interindividual susceptibility and the need for vigilance even with commonly used agents [182]. Comparative analyses in specific clinical contexts (e.g., UTI treatment) suggest that some antibiotic regimens may be associated with larger INR increases than alternatives, supporting careful selection and intensified INR monitoring when feasible [183].

Antibiotic co-prescription has also been linked to increased risk of serious bleeding events in large real-world cohorts [184]. In that cohort, early INR evaluation after co-prescription was associated with a lower risk of serious bleeding, highlighting a potentially modifiable safety practice in observational analyses [185,186]. These findings align with guideline recommendations emphasizing proactive INR surveillance and dose adjustment strategies during exposure to interacting medications, including antimicrobials [172,186]. 

### 8.3. DOACs: Plausible Microbiome Effects on Exposure, but Limited Direct Clinical Evidence 

DOACs do not depend on vitamin K cycling, yet their oral absorption and systemic exposure can be influenced by intestinal efflux transporters and metabolic pathways, particularly P-glycoprotein (P-gp) and (for some agents) cytochrome P450 3A4 (CYP3A4) [187,188,189]. Within this pharmacologic framework, the gut microbiome could plausibly influence DOAC exposure indirectly by modulating intestinal transporter expression, epithelial barrier function, and host inflammatory tone [190,191,192].

However, DOAC-specific clinical studies integrating microbiome profiling with measured DOAC concentrations and clinical outcomes remain limited, and current DOAC interaction guidance is largely derived from conventional perpetrator drugs and host factors rather than microbiome states [189,193,194]. Accordingly, microbiome–DOAC interactions should currently be framed as a biologically plausible hypothesis and an important research gap rather than an established determinant of anticoagulant response in VTE [188,189,195]. The main microbiome-related mechanisms potentially influencing anticoagulant response, together with their evidence base and possible implications for VTE care, are summarized in Table 3. Overall, the clinically strongest evidence relates to warfarin destabilization during antibiotic exposure, whereas explicit microbiome-specific effects remain difficult to isolate, and DOAC-related evidence remains sparse and hypothesis-generating.

### 8.4. Translational Implications for VTE Care 

From a translational standpoint, the microbiome–warfarin axis currently appears more clinically relevant than other proposed microbiome–anticoagulant interactions, although its practical implications remain largely indirect and supportive rather than microbiome-guided. Current evidence is most consistent with intensified safety monitoring during periods of abrupt microbiome perturbation, particularly antibiotic exposure, rather than with any formal microbiome-based adjustment strategy [172,178,184,186,196]. Considering dysbiosis as an additional contextual factor may help frame anticoagulation instability in complex patients experiencing abrupt microbiome perturbations (e.g., antibiotic exposure), although its incremental value for formal risk stratification remains unproven, and it does not currently support microbiome-based anticoagulant management [190,191,195].

For DOACs, future studies should integrate microbiome sequencing with pharmacokinetic measurements (drug concentrations and/or calibrated anti-Xa activity where applicable) and clinical outcomes to determine whether microbiome-linked modulation of transporter or metabolic pathways contributes meaningfully to exposure variability under real-world conditions [188,195,197]. Mechanistic insights into microbiome regulation of P-gp support focusing future pharmacomicrobiome studies on settings where dysbiosis is common, including antibiotic exposure, inflammatory gut states, and high polypharmacy [190,191,192]. Such work should be viewed as exploratory and aimed at defining whether any clinically actionable signal exists, rather than assuming near-term applicability to routine DOAC management. Routine microbiome testing to guide anticoagulant choice, dosing, or monitoring is therefore not supported outside research settings.

## 9. Translational Perspectives and Future Directions

Because current direct human VTE-specific evidence remains sparse, the priorities outlined below should be viewed primarily as a research agenda rather than near-term clinical applications. Prospective, well-phenotyped cohorts with serial biosampling are required to move the microbiome–VTE field from cross-sectional association toward temporal ordering and more robust causal inference, ideally capturing the pre-event baseline, acute VTE, and convalescent phases, with standardized documentation of anticoagulation, antibiotics, and dietary exposures [198,199].

Causal inference frameworks such as MR can complement cohort work, but MR outputs should be triangulated with longitudinal microbiome–metabolomics and inflammatory readouts to address pleiotropy and context dependence [163,200]. Because medication exposure is both a confounder and a potential mechanistic driver, prospective designs should also consider pharmaco-microbiome interactions, including antibiotics and other commonly used drugs that reshape microbial functions and host responses [173,201].

One possible translational direction is the exploratory development of integrated biomarker panels that could capture upstream microbial triggers and downstream thromboinflammatory effectors, rather than relying on any single marker. Circulating proxies of endotoxemia are of particular interest because they can be operationalized in cohorts and linked to the mechanistic axes discussed earlier in this review. In discovery and validation analyses, LBP was identified as a sex-stratified biomarker associated with future VTE phenotypes, supporting the feasibility of evaluating endotoxin-related parameters in risk models rather than establishing their clinical utility [3,202]. Complementary clinical evidence indicates that low-grade endotoxemia is detectable in acute pulmonary embolism and associates with a prothrombotic fibrin clot phenotype and hypofibrinolysis markers [203,204]. Experimental data further support mechanistic plausibility: circulating LPS derived from the gut microbiota augmented thrombosis in a murine stenosis-induced DVT model, and interventions that modify microbiota or barrier signaling altered DVT burden in parallel with changes in circulating LPS [205,206]. However, these findings remain experimental and do not by themselves establish a clinically actionable biomarker strategy in human VTE.

Given the importance of immunothrombosis, NET-associated parameters may represent candidate components of multi-marker panels for recurrence stratification. In mechanistic studies, extracellular DNA and NET scaffolds promote thrombus formation and serve as procoagulant platforms, whereas dismantling NETs reduces the thrombosis burden, supporting the biological plausibility for further clinical evaluation of NET-related readouts [36,207]. In a murine DVT model, NET formation preceded and promoted thrombus propagation, and NET components (including citrullinated histone H3) were detected within thrombi, reinforcing mechanistic relevance for venous disease [37,208]. Clinically, circulating citrullinated histone H3 has been shown to predict VTE risk in cancer patients, providing proof-of-concept that NET biomarkers can carry predictive information in high-risk states outside general VTE populations [152,209]. Complement activation further amplifies thromboinflammatory loops and has been shown to contribute to platelet activation and fibrin formation in venous thrombus development, supporting exploratory evaluation of complement-linked measures in advanced biomarker panels where feasible [210,211]. Currently, however, the incremental predictive value of NET- or complement-related biomarkers beyond established clinical tools remains uncertain.

To achieve clinical utility, microbiome-informed biomarkers should be evaluated alongside existing recurrence-prediction frameworks rather than in isolation. The Vienna Prediction Model provides a mature reference structure for recurrence estimation after unprovoked VTE and a practical scaffold for testing the incremental value of omics-based markers [212,213]. Time-updated D-dimer integration refined model performance and illustrates the feasibility of dynamic risk assessment—an approach conceptually aligned with serial microbiome/metabolomics sampling [212,214]. The DASH score likewise demonstrates that parsimonious clinical and laboratory models can stratify recurrence risk, providing a benchmark against which integrated microbiome-, endotoxin-, and NET-related panels could be tested [215,216]. External validation work confirms utility and limitations across age strata, highlighting why biologically grounded biomarkers may warrant evaluation as potential tools to improve discrimination in older and comorbid populations [215]. Recent prospective evaluation and recalibration efforts of the Vienna Prediction Model underscore both feasibility and the unmet need for improved identification of truly low-risk patients. In this setting, integrated microbial and thromboinflammatory panels should be explored only if they can demonstrate reproducibility, external validity, and incremental utility beyond existing recurrence models and D-dimer strategies [217,218,219].

Microbiome-targeted interventions, if investigated at all in this context, should be approached as adjunctive, mechanism-guided strategies rather than as stand-alone antithrombotic therapy, with careful attention to bleeding risk and drug interactions. One rational target is microbial TMA production: non-lethal inhibition of microbial TMA lyases with 3,3-dimethyl-1-butanol (DMB) reduces TMA/TMAO in preclinical models, illustrating a possible pathway-targeted intervention strategy [220,221]. Currently, however, this remains preclinical and does not establish relevance to VTE prevention in humans. In contrast, a single lean vegan-donor fecal microbiota transplantation in patients with metabolic syndrome, while altering microbiota composition, failed to elicit changes in TMAO production capacity or in parameters related to vascular inflammation, emphasizing that not all microbiome interventions translate into functional pathway changes in humans [222,223]. Mechanistic transplantation studies nonetheless provide additional causal support at the pathway level: defined human commensals harboring choline TMA-lyase capacity (cutC) can transmit heightened platelet reactivity and thrombosis potential in gnotobiotic models, supporting further investigation of pathway-targeted manipulation rather than purely compositional endpoints [224,225]. These data remain model-based and should not be interpreted as evidence for a clinically ready microbiome intervention in VTE. Lifestyle-related exposures, including diet and physical activity, should be accounted for in future studies because they may influence both microbiome composition and vascular-inflammatory phenotypes; however, their role as microbiome-targeted adjuncts in VTE prevention remains speculative [226,227]. Integrative analytical approaches may eventually help combine clinical, laboratory, imaging, and multi-omics data, although their practical role in microbiome-informed VTE stratification remains to be established [228,229]. Overall, future intervention-oriented work should be viewed as exploratory and focused on determining whether any reproducible, clinically actionable signal exists without compromising standard guideline-based anticoagulation.

Accordingly, the key translational priority is rigorous clinical study design: standardized stool collection and sequencing, harmonized metabolomics, prespecified thromboinflammatory end points (e.g., LBP/LPS proxies and NET biomarkers such as citrullinated histone H3 (citH3)), and anchoring to validated recurrence models to determine whether microbiome-informed panels provide incremental predictive value. Any intervention-oriented strategy should likewise be required to demonstrate a reproducible signal in prospective human studies before any role in VTE care is considered [198,230]. More broadly, host–microbial interactions across barrier organs may shape systemic inflammatory phenotypes beyond the intestine, but the relevance of these cross-organ observations to VTE remains uncertain [231,232].

## 10. Strengths and Limitations

This review provides an integrative synthesis of heterogeneous, still-limited evidence examining possible links between gut microbial dysbiosis and VTE through interconnected inflammatory, metabolic, and coagulation pathways. A major strength of the present work is its integration of multiple candidate biological axes, including endotoxin-driven TF activation; NET formation; and microbiome-derived metabolites such as TMAO, PAGln, and SCFAs, while explicitly recognizing that the strength of evidence differs across these pathways. Another strength is the incorporation of diverse lines of evidence spanning experimental models, human observational studies, and genetic approaches such as MR, as well as their interpretation with attention to their differing clinical directness and translational relevance. Integrating these complementary data sources permits a more critical appraisal of the biological plausibility and evidentiary limits of proposed links between microbial ecology and thrombotic disease. Furthermore, the discussion situates microbiome–VTE interactions within the broader landscape of chronic inflammatory disorders, including obesity, metabolic dysfunction-associated steatotic liver disease, IBD, and malignancy, thereby emphasizing shared pathophysiological pathways while distinguishing such contextual evidence from direct VTE-specific data.

However, several limitations must be acknowledged. First, the number of studies specifically investigating the gut microbiome in patients with VTE remains limited, and direct human evidence on VTE is both sparse and fragmented. Much of the mechanistic evidence derives from preclinical models or from studies conducted in the context of arterial cardiovascular disease, which may not fully reflect the biological features of venous thrombosis and therefore require cautious extrapolation. Second, substantial methodological heterogeneity exists across microbiome studies, including differences in sampling procedures, sequencing platforms, bioinformatic pipelines, metabolomic integration, and taxonomic resolution, which complicates cross-study comparisons and reproducibility. In addition, many studies rely on single-time-point sampling, which may not adequately capture the temporal dynamics of microbiome composition or metabolite exposure before, during, and after VTE events. Third, microbial composition is strongly influenced by diet, medications (including antibiotics and anticoagulants), and comorbid conditions, introducing potential confounding factors that are often difficult to control in observational cohorts. Acute illness, hospitalization, antimicrobial exposure, and anticoagulant treatment at or around the time of VTE may also distort microbiome and metabolite readouts, complicating causal interpretation and raising the possibility of reverse causation. Fourth, genetic and MR-based findings remain model-dependent and subject to the limitations of current microbiome GWAS instruments, including limited statistical power, taxonomic imprecision, and difficulty verifying core causal assumptions. Fifth, because this is a narrative review integrating heterogeneous evidence types, no formal risk-of-bias tool or quantitative synthesis was applied, which limits the ability to compare findings across studies with different designs and levels of directness to VTE. Finally, many reported associations remain indirect, exploratory, or model-based, underscoring the need for well-phenotyped prospective cohorts and mechanistic studies specifically focused on venous thrombotic disease before firm conclusions about microbiome-specific clinical relevance in VTE can be drawn or any role in routine clinical risk stratification or management can be supported.

## 11. Conclusions

Gut dysbiosis, endotoxemia, and microbiome-derived metabolites may intersect with thromboinflammatory pathways relevant to VTE, although the available support remains uneven and is strongest at the level of biological plausibility rather than direct clinical proof. Among the proposed mechanisms, the LPS–TF–NET axis represents one of the most coherent hypothesis-generating frameworks, but it is not yet established as a microbiome-specific causal pathway in human VTE. In parallel, microbiome-derived metabolites such as TMAO, PAGln, bile acid derivatives, and SCFAs may modulate platelet function, inflammatory signaling, and vascular homeostasis in experimental or non-VTE settings, whereas direct human evidence for analogous effects in VTE remains limited and, in some areas, inconsistent.

Although much of the current evidence remains indirect, associative, or derived from experimental models, emerging human, genetic, and multi-omics studies support continued investigation of the possibility that host–microbiome interactions may influence thrombotic risk, but they do not yet define a consistent or causally established signal. Future research should prioritize longitudinal and mechanistically informed human studies to clarify causality and determine whether microbiome-informed biomarkers provide reproducible incremental value beyond established VTE risk stratification models; interventional strategies should be considered only after stronger causal and translational signals emerge. Currently, however, the available evidence does not support microbiome-based risk stratification or targeted microbiome intervention as part of routine VTE care.

## Figures and Tables

**Table 1:** Dysbiosis-associated triggers, barrier dysfunction, and thromboinflammatory mechanisms potentially relevant to elevated VTE risk.

Study	Dysbiosis-Related Trigger/State	Key Host Pathway/Mediator	Proposed Thromboinflammatory Consequence Relevant to VTE	Evidence Type	Direct Relevance to VTE
Park et al. 2013 [48] Park et al. 2009 [49]	Gram-negative bacterial LPS as an innate immune trigger	LPS recognition by TLR4–MD-2 → NF-κB signaling	Innate immune activation that may prime proinflammatory and potentially procoagulant vascular and leukocyte phenotypes, providing mechanistic context	Structural/mechanistic; review synthesis	Mechanistic/contextual
Needham et al. 2013 [50] Raetz et al. 2002 [51]	Lipid A structural heterogeneity (hexa-acylated vs. underacylated LPS)	Lipid A acylation/phosphorylation tunes TLR4 activation potency	Variable inflammatory signaling intensity, with indirect implications for systemic inflammatory tone relevant to dysbiosis-associated host responses	Mechanistic review/biochemical background	Mechanistic/contextual
Levi et al. 2010 [43] Aras et al. 2004 [44] Opal et al. 2003 [32]	Endotoxin exposure (human endotoxemia)	LPS-driven TF induction and coagulation pathway engagement	In vivo activation of coagulation, including increased coagulation activation markers and TF-related activity, supporting biologic plausibility	Human experimental model; mechanistic review	Indirect human/related mechanism
Martinod et al. 2014 [35] Brill et al. 2012 [37] Fuchs et al. 2010 [36]	Inflammation-associated NET formation	NET formation as an innate effector response; NET scaffolds support thrombosis-related processes	Enhanced thrombus propagation and thrombosis susceptibility in experimental venous models, with microbiome relevance remaining indirect	Review; experimental venous model (murine DVT)	Experimental venous model
Turner 2009 [45] Groschwitz et al. 2009 [56]	Barrier dysfunction (tight junction disruption; cytokine-driven opening)	Claudins/occludin/ZO proteins; cytokine-mediated permeability increase	Increased permeability that may enable systemic translocation of microbial products, including LPS, providing indirect context for thromboinflammatory signaling	Barrier biology review; mechanistic epithelial study	Mechanistic/contextual
Fasano 2011 [58]	Zonulin-regulated permeability pathways	Zonulin-mediated modulation of intestinal barrier function	Barrier loosening potentially permissive for greater exposure to luminal microbial components, with indirect relevance to VTE-related pathways	Mechanistic review	Mechanistic/contextual
Cani et al. 2007 [46] Cani et al. 2008 [59]	High-fat-diet-associated dysbiosis and “metabolic endotoxemia”	Increased circulating LPS; microbiota-dependent endotoxemia and low-grade inflammation	Chronic low-grade inflammatory activation relevant to thromboinflammatory risk in metabolic disease, but not specific evidence for VTE	Experimental (mouse)	Mechanistic/contextual
Pussinen et al. 2011 [47] Wiedermann et al. 1999 [60]	Chronic endotoxemia proxies in populations	LBP as an indirect biomarker of endotoxin exposure	Association with systemic inflammation and cardiometabolic risk, providing indirect human context for thromboinflammatory priming	Prospective population studies	Indirect human/related disease
Ageno et al. 2008 [22] Borch et al. 2011 [62] Stein et al. 2005 [61]	Obesity/metabolic syndrome as high-risk states	Systemic inflammatory–metabolic perturbations	Increased incident VTE risk in observational epidemiology, supporting a relevant clinical context rather than a microbiome-specific mechanism	Meta-analysis; prospective cohort	Indirect human/related disease
Grainge et al. 2010 [23] Yuhara et al. 2013 [69] Yu 2018 [71]	IBD as dysbiosis-associated inflammatory disease	Dysbiosis, epithelial barrier disruption, and active inflammation	Increased VTE risk, particularly during active disease, in a dysbiosis-associated inflammatory context	Cohort; meta-analysis; microbiome review	Indirect human/related disease
Khorana et al. 2008 [73] Abdol Razak et al. 2018 [74]	Malignancy (especially during chemotherapy)	High thrombotic risk captured by prediction models; systemic inflammation	Substantial thrombosis incidence during chemotherapy, supporting a high-risk clinical setting in which thromboinflammatory pathways may be relevant	Clinical prediction model; review	Indirect human/related disease
Falanga et al. 2012 [75] Falanga et al. 2015 [24]	Cancer-associated thromboinflammation	Inflammation- and TF-linked procoagulant signaling	Amplified coagulation activation considered relevant to venous thrombogenesis, although not specific to microbiome-driven mechanisms	Mechanistic review	Indirect human/related mechanism

Evidence should be interpreted hierarchically, with direct human VTE-specific studies considered the most clinically informative, followed by human data from related diseases, while preclinical, mechanistic, and review-based evidence offers lower direct clinical relevance. Abbreviations: DVT—deep vein thrombosis; IBD—inflammatory bowel disease; LBP—lipopolysaccharide-binding protein; LPS—lipopolysaccharide; MD-2—myeloid differentiation protein 2; NET—neutrophil extracellular trap; NF-κB—nuclear factor kappa B; TF—tissue factor; TLR4—Toll-like receptor 4; VTE—venous thromboembolism; ZO—zonula occludens.

**Table 2:** Microbiome-derived metabolites implicated in thrombosis-related and thromboinflammatory signaling, along with their evidence level.

Metabolite	Microbial Pathway	Experimental Model(s)	Reported Biological Effect	Hemostatic/Inflammatory Target	Evidence Level	Study
TMAO (platelet/thrombosis axis)	Microbial conversion of choline/phosphatidylcholine to TMA → hepatic FMO3 oxidation to TMAO	Human prospective cohort; murine thrombosis model; ex vivo platelet assays	Elevated plasma TMAO associated with incident cardiovascular events in arterial/cardiometabolic cohorts; enhanced platelet Ca^2+^ signaling; accelerated thrombosis in murine models	Platelet activation; thrombosis susceptibility (primarily arterial and experimental models)	Human cardiovascular cohort + animal model + ex vivo human platelets; direct human VTE evidence limited and includes a null study	Tang et al. 2013 [114] Zhu et al. 2016 [117] Reiner et al. 2019 [116]
TMAO (vascular inflammatory axis)	As above	In vitro vascular cell studies	Activation of MAPK and NF-κB signaling in vascular cells	Vascular inflammatory signaling in non-VTE in vitro settings	In vitro mechanistic study; direct VTE relevance not established	Seldin et al. 2016 [119]
PAGln	Microbial metabolism of phenylalanine	Human cardiovascular cohort; murine thrombosis model; ex vivo platelet functional assays	PAGln levels associated with cardiovascular risk; enhanced platelet responsiveness via adrenergic receptor signaling in experimental and cardiovascular settings	Platelet responsiveness and aggregation via adrenergic receptors	Human cardiovascular cohort + animal model + ex vivo platelets; direct VTE relevance not established	Nemet et al. 2020 [132]
SCFAs (acetate, propionate, butyrate)	Fermentation of dietary fiber by gut microbiota	Murine models; in vitro immune assays	Promotion of regulatory T-cell differentiation via HDAC inhibition and GPR41/GPR43 signaling	Immune regulation and anti-inflammatory signaling	Animal model + in vitro immune studies; thrombosis relevance indirect	Smith et al. 2013 [123] Koh et al. 2016 [122]
Propionate (SCFA subtype)	As above	Murine model of hypertensive cardiovascular injury	Reduced cardiac hypertrophy and vascular dysfunction in a murine cardiovascular model	Vascular function and inflammatory modulation	Animal model; cardiovascular relevance described	Bartolomaeus et al. 2019 [125]
Secondary bile acids	Microbial biotransformation of primary bile acids	Mechanistic and biochemical studies	Modulation of FXR- and TGR5-related signaling pathways	Metabolic and inflammatory signaling pathways	Mechanistic review + experimental biochemical studies; direct VTE relevance not established	Ridlon et al., 2006 [128] Li et al. 2014 [129]
Indole derivatives	Microbial metabolism of tryptophan	Murine models; mucosal immune assays; mechanistic studies	Activation of AhR signaling; regulation of mucosal immune homeostasis	Barrier integrity and immune regulation	Animal model + mechanistic immunology studies; thrombosis relevance indirect and hypothesis-generating	Zelante et al. 2013 [134]

Abbreviations: AhR—aryl hydrocarbon receptor; Ca^2+^—calcium; FMO3—flavin-containing monooxygenase 3; FXR—farnesoid X receptor; GPR41—G protein-coupled receptor 41; GPR43—G protein-coupled receptor 43; HDAC—histone deacetylase; MAPK—mitogen-activated protein kinase; NF-κB—nuclear factor kappa B; PAGln—phenylacetylglutamine; SCFAs—short-chain fatty acids; TGR5—Takeda G protein-coupled receptor 5; TMA—trimethylamine; TMAO—trimethylamine N-oxide.

**Table 3:** Proposed microbiome-related mechanisms that may influence anticoagulant response in VTE care, together with the differing strength and clinical directness of available evidence.

Study	Drug/ Context	Microbiome- Related Mechanism	Potential Clinical Consequence	Evidence Type	Direct Clinical Relevance	Practical Implication
Olson 1984 [175] Conly et al. 1992 [176] Conly et al. 1994 [177]	Warfarin	Bacterial menaquinone production and vitamin K availability	INR variability; altered warfarin sensitivity	Mechanistic; human physiologic	Indirect/mechanistic	Consider microbiome-linked vitamin K perturbation as one possible contributor to unstable INR
Conly et al. 1994 [177] Weersma et al. 2020 [173]	Warfarin during antibiotic exposure	Antibiotic-associated dysbiosis and reduced vitamin K2 availability	Increased warfarin sensitivity; overanticoagulation risk	Mechanistic; translational review	Biologically plausible, but not microbiome-specific	Interpret post-antibiotic INR increase as potentially multifactorial, including possible microbiome effects
Glasheen et al. 2005 [179] Penning-van Beest et al. 2001 [180]	Warfarin + commonly used antibiotics	Antibiotic-associated VKA destabilization with possible microbiome contribution	Overanticoagulation	Observational; pharmacoepidemiologic	Clinically relevant, but microbiome-specific contribution difficult to isolate	Closer INR surveillance after antibiotic initiation
Tilstone et al. 1977 [181] Foster et al. 1999 [182] Saum et al. 2016 [183]	Warfarin + selected antibiotic regimens	Agent-specific antibiotic effects on INR stability	Marked INR elevation; higher bleeding susceptibility	Case reports; comparative clinical	Clinically relevant, but not specifically attributable to microbiome effects	Careful antibiotic selection and intensified INR monitoring
Lane et al. 2014 [184] Cunningham et al. 2011 [185]	Warfarin + antibiotic co-prescription	Antibiotic-associated anticoagulation destabilization in routine care	Serious bleeding events	Observational cohort	High clinical relevance, but microbiome-specific contribution uncertain	Early INR evaluation may improve safety in observational studies
Holbrook et al. 2012 [172] Ageno et al. 2012 [186]	VKA management	Interacting drugs and anticoagulant instability	Avoidable INR excursions and bleeding	Guideline-based clinical evidence	High clinical relevance, but not microbiome-specific	Proactive monitoring and dose adjustment during antimicrobial exposure
Byon et al. 2019 [187] Foerster et al. 2020 [188] Ferri et al. 2022 [189]	DOACs	Possible indirect effects via P-gp and CYP3A4-related pathways	Potential exposure variability	Pharmacokinetic reviews	Limited direct clinical evidence	No basis for microbiome-guided DOAC dose adjustment currently
Foley et al. 2021 [190] Gao et al. 2017 [191] Priyamvada et al. 2016 [192]	DOACs in dysbiosis-prone states	Possible effects on barrier integrity, transporter expression, and inflammatory tone	Hypothetical variability in absorption/exposure	Mechanistic; preclinical	Hypothesis-generating	Currently relevant mainly as a research direction
Ferri et al. 2022 [189] Sodhi et al. 2020 [193] Gronich et al. 2021 [194] Dempsey et al. 2019 [195]	DOACs: evidence gap	Limited direct microbiome–DOAC clinical evidence	Uncertain clinical significance	Review-based synthesis; indirect clinical evidence	Sparse direct evidence/research gap	Framed as hypothesis and research priority, no established clinical determinant

Interpretive note: The clinically strongest evidence in this table concerns warfarin destabilization during antibiotic exposure, although the specific contribution of microbiome disruption remains difficult to separate from conventional drug–drug interactions and other host factors. In contrast, proposed microbiome-related effects on DOAC exposure are supported mainly by pharmacokinetic, mechanistic, or preclinical evidence and remain hypothesis-generating. Abbreviations: CYP3A4—cytochrome P450 3A4; DOACs—direct oral anticoagulants; INR—international normalized ratio; P-gp—P-glycoprotein; VKA—vitamin K antagonist; VTE—venous thromboembolism.

## Data Availability

No new data were created or analyzed in this study. Data sharing is not applicable to this article.

## Supplementary Materials

The following supporting information can be downloaded at: https://www.mdpi.com/article/10.3390/nu18081231/s1. Supplementary Method S1: Full PubMed search strategy.

## Abbreviations

The following abbreviations are used in this manuscript:

ABCA1	ATP-binding cassette transporter A1
AhR	aryl hydrocarbon receptor
AI	artificial intelligence
APC	activated protein C
C3a	complement component 3a
C5a	complement component 5a
Ca^2+^	calcium
CD14	cluster of differentiation 14
citH3	citrullinated histone H3
CTEPH	chronic thromboembolic pulmonary hypertension
cutC	choline TMA-lyase capacity
CYP3A4	cytochrome P450 3A4
DMB	3,3-dimethyl-1-butanol
DNA	deoxyribonucleic acid
DOAC(s)	direct oral anticoagulant(s)
DVT	deep vein thrombosis
eNOS	endothelial nitric oxide synthase
EPCR	endothelial protein C receptor
FMO3	flavin-containing monooxygenase 3
FXR	farnesoid X receptor
GPR	G-protein-coupled receptor
GPR41	G protein-coupled receptor 41
GPR43	G protein-coupled receptor 43
GWAS	genome-wide association study
HDAC	histone deacetylase
IBD	inflammatory bowel disease
IL-1β	interleukin 1 beta
IL-6	interleukin 6
IL-8	interleukin 8
INR	international normalized ratio
IRF3	interferon regulatory factor 3
LBP	lipopolysaccharide-binding protein
LPS	lipopolysaccharide
MAPK	mitogen-activated protein kinase
MASLD	metabolic dysfunction-associated steatotic liver disease
MD-2	myeloid differentiation protein 2
MR	Mendelian randomization
MyD88	myeloid differentiation primary response 88
NET(s)	neutrophil extracellular trap(s)
NF-κB	nuclear factor kappa B
Nrf2	nuclear factor erythroid 2-related factor 2
PAGln	phenylacetylglutamine
PAI-1	plasminogen activator inhibitor 1
PAMPs	pathogen-associated molecular patterns
PAR-1	protease-activated receptor 1
PAR-4	protease-activated receptor 4
PE	pulmonary embolism
P-gp	P-glycoprotein
PSGL-1	P-selectin glycoprotein ligand-1
PTS	post-thrombotic syndrome
SCFA(s)	short-chain fatty acid(s)
TF	tissue factor
TGR5	Takeda G-protein-coupled receptor 5
TIR	Toll/interleukin-1 receptor
TLR4	Toll-like receptor 4
TMA	trimethylamine
TMAO	trimethylamine N-oxide
TNF-α	tumor necrosis factor alpha
tPA	tissue-type plasminogen activator
Treg	regulatory T cell
TRIF	TIR-domain-containing adapter-inducing interferon-β
uPA	urokinase-type plasminogen activator
VKA	vitamin K antagonist
VTE	venous thromboembolism
ZO	zonula occludens
