# Supplementary material for: Venous Thromboembolism and Gut Dysbiosis: Mechanistic Links Between Endotoxemia, Microbial Metabolites, and Thromboinflammation
Source: Nutrients. 2026 Apr 14;18(8):1231. doi: 10.3390/nu18081231 (PMC13118694; doi:10.3390/nu18081231)
Supplement: Supplementary file 1 [file nutrients-18-01231-s001.zip › nutrients-4226319-supplementary.pdf]

Supplementary Material 1

Supplementary Method S1. Full PubMed search strategy

To improve methodological transparency, the full PubMed search strategy used in this narrative review is provided below. The search was designed to identify publications addressing associations between the gut microbiome and venous thromboembolism (VTE), including deep vein thrombosis (DVT) and pulmonary embolism (PE), as well as related mechanisms involving intestinal barrier dysfunction, endotoxemia, microbiota-derived metabolites, immunothrombosis, and anticoagulant-related microbiome interactions.

The search strategy was developed using three main concept domains:

(1) microbiome-related exposure terms,
(2) VTE-related outcome terms, and
(3) mechanistic thromboinflammatory terms.

The PubMed search combined free-text terms and synonyms relevant to the scope of the review. Search terms were combined using Boolean operators (AND/OR). The strategy was intended to capture both direct VTE-specific studies and indirect mechanistic literature relevant to endothelial activation, gut barrier dysfunction, microbial translocation, coagulation, and microbiome-anticoagulant interactions. The search string shown below represents the final PubMed strategy used for this review and is aligned with the broader methodology described in the main manuscript. Comparable syntax-adapted strategies based on the same three concept domains were applied in Scopus and Web of Science.

Database: PubMed/MEDLINE
Search coverage: from database inception to February 2026
Language restriction: English
Additional database-search limits: none applied beyond the English-language restriction

Full PubMed search string:

(("gut microbiome"[Title/Abstract] OR "gut microbiota"[Title/Abstract] OR dysbiosis[Title/Abstract] OR metagenomics[Title/Abstract] OR "microbial metabolites"[Title/Abstract] OR endotoxemia[Title/Abstract] OR lipopolysaccharide[Title/Abstract] OR "intestinal permeability"[Title/Abstract] OR "gut barrier"[Title/Abstract] OR "trimethylamine N-oxide"[Title/Abstract] OR TMAO[Title/Abstract] OR "short-chain fatty acids"[Title/Abstract] OR SCFAs[Title/Abstract] OR "bile acids"[Title/Abstract])
AND
("venous thromboembolism"[Title/Abstract] OR VTE[Title/Abstract] OR "deep vein thrombosis"[Title/Abstract] OR DVT[Title/Abstract] OR "pulmonary embolism"[Title/Abstract] OR PE[Title/Abstract] OR thrombosis[Title/Abstract])
AND
("tissue factor"[Title/Abstract] OR thrombin[Title/Abstract] OR "platelet activation"[Title/Abstract] OR "neutrophil extracellular traps"[Title/Abstract] OR NETs[Title/Abstract] OR complement[Title/Abstract] OR immunothrombosis[Title/Abstract] OR coagulation[Title/Abstract]))

Following database retrieval, records were deduplicated before screening. Titles, abstracts, and full texts were assessed for eligibility according to predefined criteria focused on relevance to the microbiome-VTE axis or to mechanistic pathways considered potentially relevant to VTE. Non-English publications, reports without accessible full text, conference abstracts without sufficient usable data, letters to the editor without relevant analytical content, and articles not relevant to the scope of the review were excluded.

Eligible publications included original human, observational, translational, preclinical, and relevant genetic studies, as well as selected review articles used only to support contextual interpretation of the microbiome-thrombosis axis. Because direct VTE-specific human evidence remains sparse, studies of different designs were included to capture both clinically observed associations and biologically plausible mechanisms. However, these evidence categories were not considered equivalent and were interpreted according to study design, directness to VTE, and translational relevance.

Because this study was conducted as a narrative review rather than a systematic review, the search strategy was designed to provide a structured and transparent overview of the literature while accommodating heterogeneous evidence types, including direct human studies, translational and preclinical studies, and mechanistic literature. Accordingly, the evidence was synthesized narratively, with emphasis placed on the strength, directness, and clinical relevance of the available evidence rather than on formal quantitative comparison.
